# Refractive Index Sensing with D-Shaped Plastic Optical Fibers for Chemical and Biochemical Applications

**DOI:** 10.3390/s16122119

**Published:** 2016-12-13

**Authors:** Filipa Sequeira, Daniel Duarte, Lúcia Bilro, Alisa Rudnitskaya, Maria Pesavento, Luigi Zeni, Nunzio Cennamo

**Affiliations:** 1 Instituto de Telecomunicações, 3810-193 Aveiro, Portugal; dduarte@ua.pt (D.D.); lucia.bilro@av.it.pt (L.B.); 2 CESAM, University of Aveiro, 3810-193 Aveiro, Portugal; alisa@ua.pt; 3 I3N/FSCOSD, Department of Physics, University of Aveiro, 3810-193 Aveiro, Portugal; 4 Department of Chemistry, University of Aveiro, 3810-193 Aveiro, Portugal; 5 Department of Chemistry, University of Pavia, 27100 Pavia, Italy; maria.pesavento@unipv.it; 6 Department of Industrial and Information Engineering, Second University of Naples, 81031 Aversa, Italy; zeni@unina.it (L.Z.); nunzio.cennamo@unina2.it (N.C.)

**Keywords:** plastic optical fiber (POF), refractive index sensors, optical fiber sensors, remote sensing, chemical and biochemical sensing

## Abstract

We report the optimization of the length of a D-shaped plastic optical fiber (POF) sensor for refractive index (RI) sensing from a numerical and experimental point of view. The sensing principle is based on total internal reflection (TIR). POFs with 1 mm in diameter were embedded in grooves, realized in planar supports with different lengths, and polished to remove the cladding and part of the core. All D-shaped POF sensors were tested using aqueous medium with different refractive indices (from 1.332 to 1.471) through intensity-based configuration. Results showed two different responses. Considering the refractive index (RI) range (1.33–1.39), the sensitivity and the resolution of the sensor were strongly dependent on the sensing region length. The highest sensitivity (resolution of 6.48 × 10^−3^ refractive index units, RIU) was obtained with 6 cm sensing length. In the RI range (1.41–1.47), the length of the sensing region was not a critical aspect to obtain the best resolution. These results enable the application of this optical platform for chemical and biochemical evanescent field sensing. The sensor production procedure is very simple, fast, and low-cost.

## 1. Introduction

In recent years, plastic optical fibers (POFs) have been well known for their successful use as optical fiber sensors (OFS) [1,2,3]. These fibers share some attributes with glass optical fibers (GOF), such as immunity to electromagnetic fields, small size and weight, but also allow the production of low-cost sensing systems. Compared to GOFs, POFs show higher resistance to harsh environments, flexibility, simpler manufacturing and handling procedures, great numerical aperture, and allow easy connectorization due to the large diameter of the fibers.

Bilro et al. presented a review of POF sensor technology with a special focus on intensity variation schemes and low-cost solutions [4]. Jin and Granville reviewed the recent progress in POF sensors, focusing on intrinsic detection schemes [5]. OFSs can be classified as intrinsic and extrinsic, depending on whether the fiber is interacting with an analyzed medium or if it is used only as a waveguide that allows the propagation of the light to the sensing region, respectively. Further, the detection scheme can be based on reflexion, where the light source and detector are placed on the same side of the fiber; or on transmission, if they are placed on opposite sides. In both detection schemes, there are several transduction mechanisms that can be employed, including sensors based on variations of the evanescent field or in spectroscopic methods (absorption, fluorescence, and refractive index, RI). The light is propagated in an optical fiber by total internal reflection (TIR), and in a standard optical fiber, the interaction of the evanescent field with the external medium is negligible (radiation penetrates into the fiber cladding, with penetration depth around hundreds of nanometers, and whose energy decays exponentially with the distance from the core–cladding interface). For an optical fiber to be used as a sensor, to detect changes in the external medium—such as variations of the external refractive index or changes occurring in a selective layer deposited on the fiber—the light that propagates in the fiber core should interact directly or be able to sense the variations occurring in the external medium or in the selective layer. One simple procedure is to partially or totally remove the cladding of the fiber where the core has to be exposed. This can be done by applying mechanical tapering or chemical etching, where the cladding is removed along the thickness of the fiber. The cladding can also be removed by side-polishing on one side of the fiber, and a D-shape can be obtained. Depending on the aim of the application, one can choose to remove only the cladding (partially or totally) or also part of the core. In a fiber with an exposed core, the external medium acts as a “substitute” cladding, and the light that propagates in the fiber will interact with this external medium, and changes of RI can be monitored. When the aim is chemical sensing by the use of selective layers, this layer will be the substitute cladding, and the changes that occur in this layer can also be detected and monitored; for example, by the use of molecularly imprinted polymers (MIPs), where the binding of the template molecule with the MIP causes a variation in the polymer matrix. 

Several studies related to RI-POF sensors can be found in the literature. Bilro et al. reported theoretical modelling of D-shaped POFs at different macrobending conditions and external RI, which was validated by experimental results [6]; the experimental study was performed with different conditions of macrobending and external RI for sensing regions with lengths of 1.3 cm, 1.5 cm, and 2.1 cm with a total depth of 550 μm, 640 μm, and 550 μm, respectively (total depth is the total thickness of the remaining core). Feng et al. studied evanescent field sensing with the refractive index of a tapered POF at the wavelengths 532 nm, 633 nm, and 780 nm with different tapered waist diameters. This work reported that the reduction of the diameter of a tapered POF and the increase of the number of tapered regions improves the sensitivity and linearity of the sensor response. The best performance of the tapered POF based RI sensors was achieved at 633 nm for RI ranging from 1.33 to 1.41 [7]. An optimization of depth and curvature radius of a D-shaped POF with 1 cm length, aiming to increase linearity range and sensitivity to RI (1.333–1.455) at 652 nm, was also reported by Feng et al., with the best results being obtained for a depth of 500 μm and a curvature radius of 5 cm [8]. Nevertheless, a further optimization scheme was reported by the same authors—Liu and Feng, obtaining the best results using a D-shape with 2 cm length and an excurvation structure, with the same conditions of bending radius and depth; however, sensitivity and resolution of the sensor were not specified [9]. A resolution of 10^−3^–10^−4^ RIU (refractive index units) for RI range (1.333–1.403) was reported for a multi-D-shaped sensor by Chen et al.; the D-shaped regions (from three to seven, spaced 1 mm apart) were written by femtosecond laser pulses in a communication-grade multimode silica optical fiber (Corning 62.5/125 μm) with 100 μm depth, 250 μm width, and 1 mm length [10]. The transmitted power increased linearly when the sensor is exposed to sucrose solutions of increasing refractive index, although a complex procedure and expensive instrumentation are required for the sensor production. Cennamo et al. have reported several biological and chemical sensors based on SPR (surface plasmon resonance) in a D-shaped POF platform [11,12,13] with 1 cm length sensing region and with typical optical resolution of around 6 × 10^−4^ RIU [14].

In all these reported works, the length of the sensing region was not taken into account in the optimization of a sensor’s sensitivity to RI. The novelty of this work is the study of the sensitivity to RI and resolution of D-shaped POF sensors with the length of the sensing region using a low-cost procedure and an intensity-based detection scheme. Numerical and experimental results will be presented towards the optimization of the length of the D-shaped sensor for RI ranging from 1.332 to 1.471. Finally, this work reports the development of low-cost intensity-based POF sensors with resolution suitable for chemical and biochemical applications [15,16].

## 2. Materials and Methods

Commercially available plastic optical fibers (POFs) from Asahi Kasei (DB-1000, Plastic Optical Fiber Marketing & Development Group, Tokyo, Japan) were selected with the following characteristics: a polymethyl methacrylate (PMMA) core of 980 μm, a fluorinated polymer cladding of 10 μm, a numerical aperture of 0.5. The refractive index in the visible range of interest is about 1.49 for PMMA and 1.40 for the fluorinated polymer.

Glycerin was purchased from Carlo Erba Reagenti, and the solutions were prepared using deionized water. The refractive index of the tested solutions was measured in all experiments using an Abbe Refractometer, Model RMI, from Exacta and Optech Labcenter.

### 2.1. Sensors’ Development

**Figure 1:**
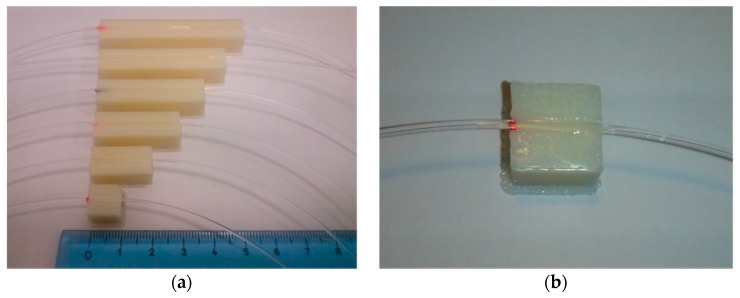
D-shaped sensors: (**a**) lengths of the sensing region ranging from 1 cm (Sensor 1) to 6 cm (Sensor 6); (**b**) Close-up of Sensor 1, with sensing region of 1 cm length.

The fibers were cut to about 20 cm length using a fiber optic cutter from Rennsteig, and embedded in grooves on planar supports (with different lengths from 1 cm to 6 cm), as shown in Figure 1.

The planar supports used in this study were made with ABS plus*™* production-grade thermoplastic, with an instrumental error of 100 μm, produced by Mojo^®^ 3D Printer (provided by Stratasys^®^ FDM^®^, Eden Prairie, MN, USA). The dimensions of the grooves were 1 mm width, 700 μm depth, and lengths from 1–6 cm (with 1 cm step). 

**Figure 2:**
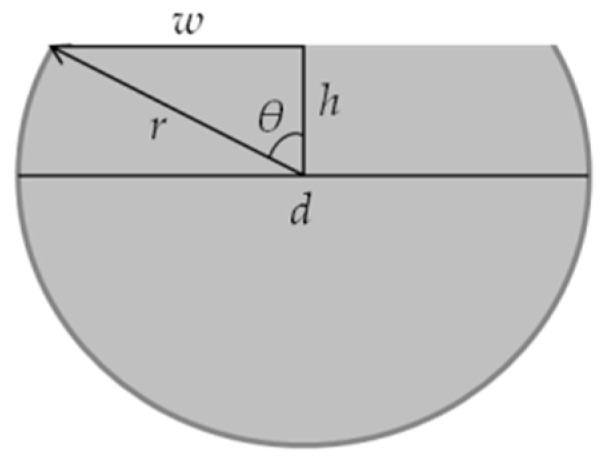
Image of a D-shaped fiber, where *d* is the fiber diameter, *r* the radius, *h* the depth, and *w* the half width of the sensing region.

The D-shaped sensors were obtained by polishing the fibers embedded in the planar support with sandpaper of 5 μm (LFG5P), 3 μm (LFG3P), and 1 μm (LFG1P), with a “figure eight” pattern. When the surface of the fibers was polished into a D-shape, the cladding and part of the core was removed (see Figure 2). The depth of the produced sensors (*h*) can be easily calculated through the fiber diameter, *d*, and the half width of the sensing region, *w*.

The depth of the fiber, *h*, is therefore calculated by simple trigonometric equations:

(1) $$h=r.\;\cos\left[\sin^{-1}\left(\frac{w}{r}\right)\right],$$

where:

(2) $$\sin\theta =\;\frac{w}{r},\;\mathrm{and}$$

(3) $$\cos\theta =\;\frac{h}{r}$$

**Figure 3:**
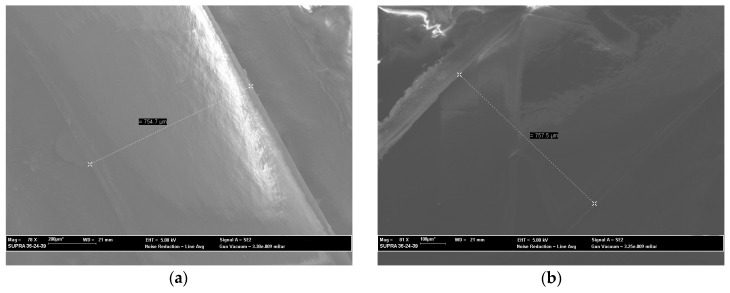
SEM images of the D-shaped sensors: (**a**) Sensor 2; (**b**) Sensor 5.

The D-shaped POF region was evaluated by scanning electron microscopy (SEM, model Zeiss SUPRA35, Berlin, Germany). Figure 3 shows the analysis of the sensing area for three of the six sensors. The width of the sensing region obtained, *2w*, is around 760 μm, equivalent to a depth, *h*, of 325 μm (Equation (1)).

### 2.2. Optical Sensing Configuration

**Figure 4:**
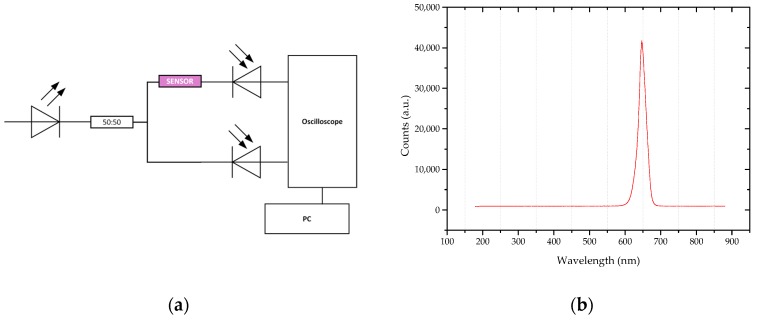
(**a**) Outline of the experimental setup; (**b**) LED spectrum.

Sensing in an intensity-based configuration allows for the measurement of the transmitted light that passes through the sensing area. The experimental setup (as shown in Figure 4a) comprised a stabilized power supply, LED (Avago SFH757V, Broadcom Limited, San Jose, CA, USA), wavelength centered at 650 nm, see Figure 4b), an optical coupler (50:50), two photodetectors (Avago SFH250V, Broadcom Limited, San Jose, CA, USA), and an oscilloscope (Picoscope). Output data, time, and voltage of the reference and sensor signals—in mV (*V_reference_* and *V_sensor_*, respectively)—were logged into a PC by means of Picoscope’s software. The self-referenced transmitted signal (*k*) was used to correct source fluctuations and variations due to external conditions, as shown in Equation (4):

(4) $$k=\frac{V_{sensor}}{V_{reference}}$$

After adding the test solution to the D-shaped sensor, the signals were recorded for 5 min, and the mean value of the referenced signal (*k*) with respective mean absolute relative error (MARE) was calculated with MATLAB software.

### 2.3. Refractive Index Measurements

All the D-shaped POF sensors were tested using glycerin solutions with increasing refractive index varying from 1.332 to 1.471. In each performed test, the refractive index of the tested solutions was measured with an Abbe Refractometer.

Each test started by adding deionized water to the D-shaped sensor. Measurements in water (which had the lowest refractive index) were used for signal normalization, according to Equation (5):

(5) $$k_{norm}=\frac{k_{solution}}{k_{water}}$$

The D-shaped sensor was washed twice using the next test solution, with higher refractive index, to clean the surface and eliminate any residues of the previous solution. The new test solution was then added, and the signals recorded (for 5 min). This procedure was used for all the tests and all sensors.

Three replicated measurements of the sensor response to refractive index (*n_ext_*) were performed to validate the obtained results. For each sensor, the mean value of the three tests (*k_avg_*) and the respective standard deviation (*δk_avg_*) were obtained. The sensitivity (*S*) of the D-shaped sensor is defined by:

(6) $$S=\left|\frac{\partial k_{avg}}{\partial n_{ext}}\right|$$

The resolution (*Δn*) is defined as the minimum amount of change in refractive index that can be detectable, and can be defined as:

(7) $$\Delta n=\frac{1}{S}.\delta k_{avgmax}$$

where *δk_avgmax_* is the maximum value of standard deviation obtained at the refractive index range of interest.

### 2.4. Sensor Modeling

A simulation model of the sensor based on the model developed in reference [6] was implemented and compared to the experimental results. For this model, general considerations must be noted: radiation losses in multimode POFs are usually calculated by a geometric approach; light is treated as individual rays, and in this case, losses occur by the interactions of light rays with the interface layers of the fiber core and external medium through refraction and reflection; uniform mode distribution (UMD) was assumed; only meridional rays were taken into account due to their significant contribution to radiation losses; the LED light source was approximated to a Lambertian emitter, that is *I(θ) = I_0_.*cos*(θ)*, where *θ* is the angle with respect to the fiber axis and *I_0_* is the intensity for normal incidence; the polished surface will be considered as a macrobending, and its effects will also be modeled. The polished surface can be represented by an arc of a circle (concave surface) with length *L* and radius *R_b_*_0_ obtained by:

(8) $$R_{b0}=-\;\frac{L^{2}+4{\left(r-h\right)}^{2}}{8\left(r-h\right)}.$$

This bent surface will influence the power losses, being the bend angle (*θ_b_*_0_) given by:

(9) $$\theta_{b0}=\theta_{c}\sqrt{\left(1-\frac{2r}{R_{b0}\theta_{c}^{2}}\right)},$$

where *θ_c_* is the complementary angle of the critical angle for the core/cladding interface.

The light rays will encounter the side-polished interface, and a fraction of energy will be lost by transmission. The loss reflection coefficient *R_n_* can be calculated by the Fresnel’s equations for unpolarized light, through the amplitude reflection coefficients of the perpendicular and parallel polarization:

(10) $$R_{n}\left(\theta \right)=\frac{R_{⊥}^{2}+R_{∥}^{2}}{2}$$

respectively,

(11) $$R_{⊥}\left(\theta \right)=\left|\frac{n_{cr}\cos\theta -n_{ext}\sqrt{1-{\left(\frac{n_{cr}}{n_{ext}}\sin\theta \right)}^{2}}}{n_{cr}\cos\theta +n_{ext}\sqrt{1-{\left(\frac{n_{cr}}{n_{ext}}\sin\theta \right)}^{2}}}\right|\text{ and }R_{∥}\left(\theta \right)=\left|\frac{n_{cl}\sqrt{1-{\left(\frac{n_{cr}}{n_{ext}}\sin\theta \right)}^{2}-n_{ext\;}\cos\theta }}{n_{cl}\sqrt{1-{\left(\frac{n_{cr}}{n_{ext}}\sin\theta \right)}^{2}+n_{ext}\cos\theta }}\right|$$

with *n_cr_* and *n_cl_* as the fiber core and cladding refractive indices, respectively. Multiple reflections of a ray will occur over the whole sensitive section. The total number of internal reflections *N(θ)* that the light ray undergoes is the near integer obtained by

(12) $$N\left(\theta \right)=\frac{L\;\tan\theta }{2\left(r+h\right)}.$$

To simulate the roughness of the sensing region, a dependent external refractive index power loss roughness coefficient (*R_s_*) was considered. This coefficient has a linear decrease profile until reaching the fiber cladding refractive index, and it is given by:

(13) $$R_{s}\left(n_{ext}\right)=\left(\frac{s}{n_{H2O}^{2}+n_{cl}^{2}}\right)(n_{ext}^{2}+n_{cl}^{2}),$$

where *s* stands for a roughness constant that characterizes the type of polish used, and is constant to all presented sensors in this work. The normalized transmitted output of the fiber will then be calculated by:

(14) $$\eta_{RI}=\frac{P_{n_{ext}}}{P_{n_{H2O}}}=\frac{R_{s}\left(n_{ext}\right)\int_{0}^{\theta_{b0}}R_{n_{ext}}^{N\left(\theta \right)}\cos\theta \sin\theta \;d\theta }{R_{s}\left(n_{H2O}\right)\int_{0}^{\theta_{c}}R_{n_{H2O}}^{N\left(\theta \right)}\cos\theta \sin\theta \;d\theta }.$$

## 3. Results and Discussion

### 3.1. Analysis of Sensitivity and Resolution vs. Length of D-Shaped POF Region

**Figure 5:**
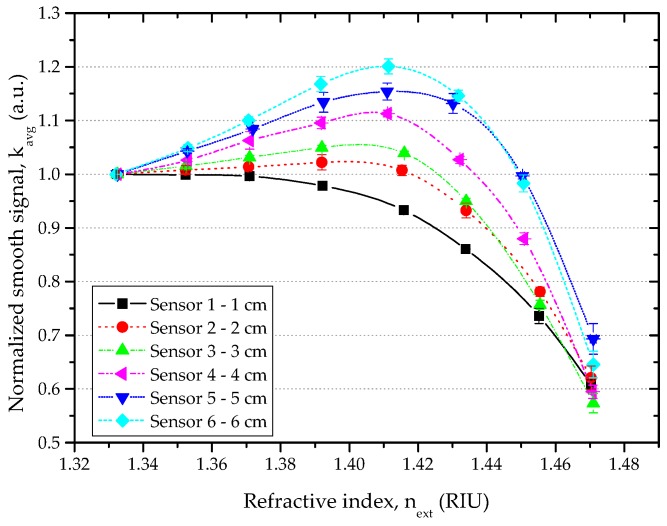
Normalized smooth signal vs. refractive index (RI) for all D-shaped sensors, RI range 1.33–1.47.

Figure 5 shows the normalized smooth signal (*k_avg_*) versus the refractive index (*n_ext_*) as measured for all the investigated sensors. The symbols represent the experimental results, and the error bars represent the standard deviation. As shown in Figure 5, the response of the D-shaped sensors to refractive index (RI) is dependent on the length of the sensing region.

**Figure 6:**
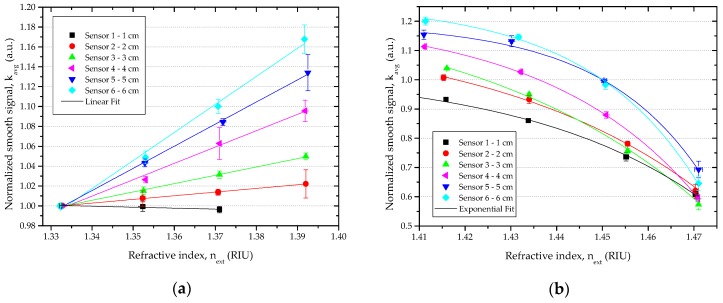
Normalized smooth signal vs. refractive index: (**a**) RI: 1.33–1.39, linear fitting; (**b**) RI: 1.41–1.47, exponential fitting.

Two different responses were observed, and are depicted in Figure 6. For the D-shaped sensor with 1 cm length (Sensor 1), the normalized smooth signal had a slight decrease in RI range between (1.33–1.37) RIU, and an exponential decrease in the refractive index range of (1.37–1.47) RIU. For all other D-shaped sensors, the normalized smooth signal increased linearly with increasing RI in the range of (1.33–1.39) RIU, and an exponential decrease of the normalized smooth signal with increasing RI was observed between (1.41–1.47) RIU.

**Figure 7:**
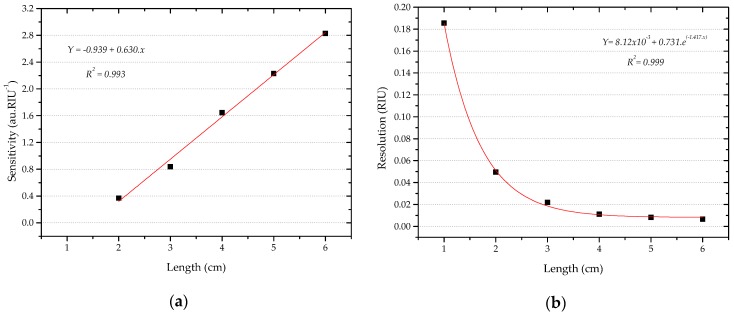
RI: 1.33–1.39: (**a**) Sensitivity and (**b**) resolution of the D-shaped sensors vs. length of the sensing region. For the D-shaped sensor with 1 cm length (Sensor 1), the resolution obtained is for the refractive index range 1.33–1.37 RIU.

The sensitivity and the resolution of the D-shaped sensors were calculated by Equations (6) and (7). The obtained results are shown in Table 1 and Figure 7 for refractive index ranging from 1.33 to 1.39 RIU. In this range, the sensitivity is the absolute value of the slope of the linear fitting obtained for each sensor.

For Sensors 2 to 6, a linear increase of the normalized smooth signal with the increase of external refractive index was observed from 1.33 to 1.39 RIU. In this refractive index range, the sensitivity and resolution of the D-shaped sensors were strongly dependent on the length of the sensing region. The sensitivity increased and the resolution decreased with increasing the length. The best results—sensitivity of 2.8271 au.RIU^−1^ and resolution of 6.48 × 10^−3^ RIU—were obtained for Sensor 6 with 6 cm sensing length (see Table 1).

**Figure 8:**
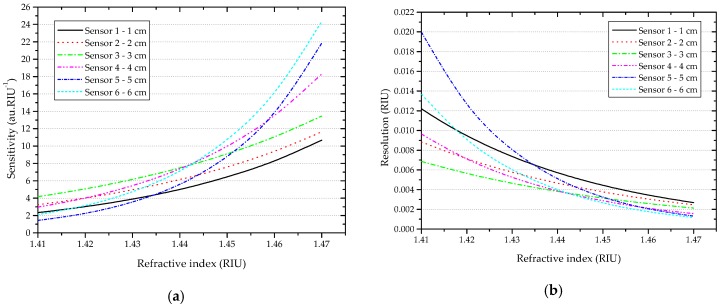
RI: 1.41–1.47: (**a**) Sensitivity and (**b**) resolution of the D-shaped sensors vs. length of the sensing region.

Sensitivity and resolution in the refractive index range from 1.41 to 1.47 RIU—where an exponential decrease of the normalized signal was observed—are shown in Table 2 and Figure 8. In this range, the sensitivity is given by the following expression:

(15) $$S_{\left(RI:1.41-1.47\right)}=\;\left|R_{0}.A.e^{R_{0}.n_{ext}}\right|.$$

Considering the refractive index range from 1.41 to 1.47 RIU, the sensitivity and resolution of the D-shaped sensors were dependent on the refractive index. In this case, the length of the sensing area was not critical to obtain the best resolution. Resolution of about 10^−3^ RIU was obtained for refractive index of 1.44 or higher for all sensors. For example, for Sensor 3 with 3 cm sensing length, a resolution of 6.86 × 10^−3^ RIU (for 1.41 RIU) and 2.13 × 10^−3^ RIU (for 1.47 RIU) was obtained, with a sensitivity of 4.1709 au.RIU^−1^ and 13.4548 au.RIU^−1^, respectively—much higher than the values obtained in the RI range from 1.33 to 1.39 RIU.

### 3.2. Comparison between Simulation and Experimental Results

**Figure 9:**
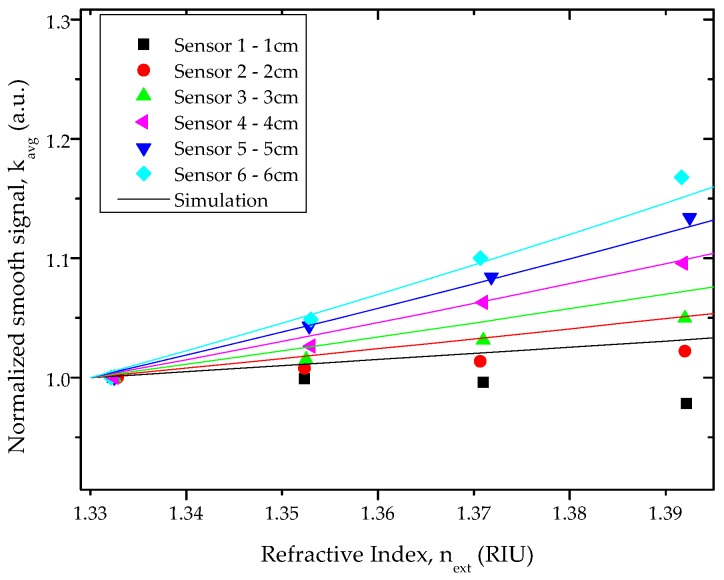
Experimental and simulation results: normalized smooth signal vs. refractive index for all D-shaped sensors in the RI range (1.33–1.39).

The model described in Section 2.4 was applied to RI from 1.33 to 1.39, a range of interest for chemical and biochemical applications. The results obtained with the simulations (solid lines) are depicted in Figure 9 and compared with the experimental results (symbols).

In the RI range (1.33–1.39), a good correlation was observed between experimental results and the simulations performed for the D-shaped sensors with length equal to or greater than 4 cm. For the sensors with shorter D-shape length, the normalized transmitted signal predicted by the model was higher than the observed one. This discrepancy between the simulated and the experimental data is possibly due to small variations in the morphology of the D-shape region (roughness and total depth) resulting from the manual process used for the preparation of the sensors.

## 4. Conclusions

D-shaped POF refractive index sensors with different sensing region lengths have been characterized. Numerical and experimental results have shown that the resolution of this optical platform is sufficiently low to enable further developments towards chemical and biochemical sensing, while also allowing low volume sampling if equipped with an appropriate flow cell. The developed D-shaped sensors are easy to produce and allow a fast and low-cost sensing. The working refractive index range is of extreme importance in order to choose the best platform with the best performance in terms of sensitivity and resolution. The refractive index range from 1.33 to 1.39 RIU is of particular interest for chemical and biochemical applications. In this range, the sensitivity and the resolution strongly depend on the length of the sensing region, with the highest sensitivity (and lowest resolution of 6.48 × 10^−3^ RIU) being obtained for the sensor with 6 cm sensing length. In the RI range from 1.41 to 1.47 RIU, the sensitivity and resolution of the sensors depend on the refractive index of interest. In this region, the selected length of the sensing region in order to obtain the best performance should take the working refractive index into account. 

The biggest innovation achieved was the development of a POF sensor for RI sensing with 6.48 × 10^−3^ RIU resolution with simple and low-cost methods, and verification of the length-dependence of the sensing region in a D-shaped POF platform, for refractive index range 1.33–1.39. A linear response was obtained for all sensors with sensing length equal to 2 cm or higher. Although the obtained resolution is lower than the one obtained for a D-shaped POF-SPR platform (6 × 10^−4^ RIU [14]), it is the same order of magnitude as the one obtained for the sensor developed in reference [10] (10^−3^–10^−4^ RIU), but without the need of expensive and complex instrumentation.

## Figures and Tables

**Table 1:** RI: 1.33–1.39—Sensitivity and resolution of the D-shaped sensors, given by Equations (6) and (7).

Sensor	Length/cm	Sensitivity	*δk_avgmax_*	Resolution	R^2^ (Linear Fit)	RI Range
Sensor 1	1	0.0987	0.0183	1.86 × 10^−1^	0.7787	1.33–1.37
Sensor 2	2	0.3699		4.95 × 10^−2^	0.9978	1.33–1.39
Sensor 3	3	0.8392		2.18 × 10^−2^	0.9982	
Sensor 4	4	1.6467		1.11 × 10^−2^	0.9902	
Sensor 5	5	2.2292		8.21 × 10^−3^	0.9986	
Sensor 6	6	2.8271		6.48 × 10^−3^	0.9917	

**Table 2:** RI: 1.41–1.47—Obtained parameters given by Equations (7) and (15), that allow the resolution and sensitivity of the D-shaped sensors to be calculated, respectively.

Sensor	Length/cm	R_0_	A	*δk_avgmax_*	R^2^ (Exponential Fit)	RI Range
Sensor 1	1	25.25	−3.19 × 10^−17^	0.0286	0.9980	1.37–1.47
Sensor 2	2	21.31	−1.36 × 10^−14^		0.9968	1.41–1.47
Sensor 3	3	19.52	−2.38 × 10^−13^		0.9917	
Sensor 4	4	30.30	−2.76 × 10^−20^		0.9995	
Sensor 5	5	45.41	−4.91 × 10^−30^		0.9882	
Sensor 6	6	40.91	−4.52 × 10^−27^		0.9916	

## References

[1] Gowri A., Sai V.V.R. (2016). Development of LSPR based U-Bent Plastic Optical Fiber Sensors. Sens. Actuators B Chem..

[2] Al-Qazwini Y., Noor A.S.M., Al-Qazwini Z., Yaacob M.H., Harun S.W., Mahdi M.A. (2016). Refractive index sensor based on SPR in symmetrically etched plastic optical fibers. Sens. Actuators A Phys..

[3] Park J., Park Y.J., Shin J.-D. (2015). Plastic optical fiber sensor based on in-fiber microholes for level measurement. Jpn. J. Appl. Phys..

[4] Bilro L., Alberto N., Pinto J.L., Nogueira R. (2012). Optical sensors based on plastic fibers. Sensors.

[5] Jin Y., Granville A.M. (2016). Polymer Fiber Optic Sensors—A Mini Review of their Synthesis and Applications. J. Biosens. Bioelectron..

[6] Bilro L., Alberto N.J., Sá L.M., Pinto J.L., Nogueira R. (2011). Analytical Analysis of Side-Polished Plastic Optical Fiber as Curvature and Refractive Index Sensor. J. Light. Technol..

[7] Feng D., Liu G., Liu X.-L., Jiang M.-S., Sui Q.-M. (2014). Refractive index sensor based on plastic optical fiber with tapered structure. Appl. Opt..

[8] Feng D., Zhang M., Liu G., Liu X.-L., Jia D.-F. (2014). D-Shaped Plastic Optical Fiber Sensor for Testing Refractive Index. IEEE Sens. J..

[9] Liu G., Feng D. (2016). Evanescent wave analysis and experimental realization of refractive index sensor based on D-shaped plastic optical fiber. Opt.-Int. J. Light Electron Opt..

[10] Chen C.H., Tsao T.C., Tang J.L., Wu W.T. (2010). A multi-D-shaped optical fiber for refractive index sensing. Sensors.

[11] Cennamo N., Varriale A., Pennacchio A., Staiano M., Massarotti D., Zeni L., D’Auria S. (2013). An innovative plastic optical fiber-based biosensor for new bio/applications. The case of celiac disease. Sens. Actuators B Chem..

[12] Cennamo N., D’Agostino G., Pesavento M., Zeni L. (2014). High selectivity and sensitivity sensor based on MIP and SPR in tapered plastic optical fibers for the detection of L-nicotine. Sens. Actuators B Chem..

[13] Cennamo N., Pesavento M., Lunelli L., Vanzetti L., Pederzolli C., Zeni L., Pasquardini L. (2015). An easy way to realize SPR aptasensor: A multimode plastic optical fiber platform for cancer biomarkers detection. Talanta.

[14] Cennamo N., Massarotti D., Conte L., Zeni L. (2011). Low cost sensors based on SPR in a plastic optical fiber for biosensor implementation. Sensors.

[15] Wang X.D., Wolfbeis O.S. (2013). Fiber-Optic Chemical Sensors and Biosensors (2008–2012). Anal. Chem..

[16] Wang X.D., Wolfbeis O.S. (2016). Fiber-Optic Chemical Sensors and Biosensors (2013–2015). Anal. Chem..

